# Prognostic value of COL10A1 and its correlation with tumor-infiltrating immune cells in urothelial bladder cancer: A comprehensive study based on bioinformatics and clinical analysis validation

**DOI:** 10.3389/fimmu.2023.955949

**Published:** 2023-03-17

**Authors:** Xiaoming Wang, Yunjin Bai, Facai Zhang, Dengxiong Li, Kai Chen, Ruicheng Wu, Yin Tang, Xin Wei, Ping Han

**Affiliations:** Department of Urology/Institute of Urology, West China Hospital, Sichuan University, Chengdu, Sichuan, China

**Keywords:** COL10A1, urothelial bladder cancer, prognosis, bioinformatics, tumor microenvironment, tumor-infiltrating immune cell

## Abstract

**Introduction:**

Bladder cancer (BLCA) is one of the most lethal diseases. COL10A1 is secreted small-chain collagen in the extracellular matrix associated with various tumors, including gastric, colon, breast, and lung cancer. However, the role of COL10A1 in BLCA remains unclear. This is the first research focusing on the prognostic value of COL10A1 in BLCA. In this research, we aimed to uncover the association between COL10A1 and the prognosis, as well as other clinicopathological parameters in BLCA.

**Methods:**

We obtained gene expression profiles of BLCA and normal tissues from the TCGA, GEO, and ArrayExpress databases. Immunohistochemistry staining was performed to investigate the protein expression and prognostic value of COL10A1 in BLCA patients. GO and KEGG enrichment along with GSEA analyses were performed to reveal the biological functions and potential regulatory mechanisms of COL10A1 based on the gene co-expression network. We used the “maftools” R package to display the mutation profiles between the high and low COL10A1 groups. GIPIA2, TIMER, and CIBERSORT algorithms were utilized to explore the effect of COL10A1 on the tumor immune microenvironment.

**Results:**

We found that COL10A1 was upregulated in the BLCA samples, and increased COL10A1 expression was related to poor overall survival. Functional annotation of 200 co-expressed genes positively correlated with COL10A1 expression, including GO, KEGG, and GSEA enrichment analyses, indicated that COL10A1 was basically involved in the extracellular matrix, protein modification, molecular binding, ECM-receptor interaction, protein digestion and absorption, focal adhesion, and PI3K-Akt signaling pathway. The most commonly mutated genes of BLCA were different between high and low COL10A1 groups. Tumor immune infiltrating analyses showed that COL10A1 might have an essential role in recruiting infiltrating immune cells and regulating immunity in BLCA, thus affecting prognosis. Finally, external datasets and biospecimens were used, and the results further validated the aberrant expression of COL10A1 in BLCA samples.

**Conclusions:**

In conclusion, our study demonstrates that COL10A1 is an underlying prognostic and predictive biomarker in BLCA.

## Introduction

Bladder cancer (BLCA) is the twelfth most common cancer worldwide, with 573,278 new cases and 212,536 deaths reported in 2020 (1). BLCA can present as non-muscle-invasive BLCA (NMIBC), muscle-invasive BLCA (MIBC), and metastatic disease. Radical cystectomy remains the standard treatment for MIBC; platinum-based chemotherapy is still the first-line chemotherapy for metastatic tumors (2). There has been no breakthrough in the treatment of BLCA over the past three decades until immune checkpoint inhibitors, fibroblast growth factor receptor (FGFR) inhibitors, and antibody-drug conjugate (ADC) targeting Nectin-4 were approved for advanced BLCA, however, overall response rates of which were less than 50% and complete response rates were less than 15% (3–5). Thus, there is a pressing need to explore prognostic and druggable biomarkers to improve the survival outcome of patients with metastatic BLCA. New biomarkers can be combined with existing new technologies, such as radiomics, to open up new clinical application-oriented research directions in the field of tumor diagnosis and treatment (6, 7).

The tumor microenvironment (TME) contains tumor cells, vasculature, extracellular matrix (ECM), stromal, and immune cells (8). ECM plays a vital role in tumor establishment, disease progression, and modulating therapeutic efficacy. ECM-related genes can be used as prognostic factors for the prognosis and recurrence of BLCA (9). Collagen, the major component of the ECM that participates in cancer fibrosis, influences cancer cell behavior. Cancer cells reversely reshape collagen to promote cancer progression (10). Collagen companies macrophages, mast cells, lymphocytes, and fibroblasts regulate cancer immunity and progression (11). Numerous clinical researches have identified collagen as a prognostic factor (10). Collagen is also associated with resistance to chemotherapy and targeted drugs in cancers (12–14). As collagen has evident genetic and epigenetic stability and is basically expressed in multiple forms of cancer, collagen can also act as a drug convener or a therapeutic target.

Type X collagen gene (COL10A1) belongs to the collagen family, which is secreted small-chain collagen and plays a vital role in the extracellular matrix (15). The function and expression level of type X collagen is affected by receptors, such as DDR2, and multiple molecular mechanisms (16, 17). Higher expression of COL10A1 protein has been revealed in cancerous tissue and has been verified to be linked with tumor angiogenesis across various types of cancer (18). COL10A1 was highly expressed in the plasma in gastric, colon, breast, and lung cancer and might be a potential diagnostic predictor (19–24). COL10A1 and the immune microenvironment can also be used as prognostic predictors of neoadjuvant therapy for breast cancer (25). Furthermore, data from *in vitro* and *in vivo* studies showed that COL10A1 promotes invasion and metastasis in gastric cancer *via* epithelial-mesenchymal transition and TGF-β signaling (26, 27). However, no previous studies have reported the role and function of COL10A1 in BLCA.

This article focused on the expression, prognostic, and immune implications of COL10A1 in BLCA. Data from The Cancer Genome Atlas (TCGA) and the Gene Expression Omnibus (GEO) database were downloaded and mined to evaluate the role of COL10A1 in BLCA. Bioinformatics analyses confirmed the expression profile and prognostic value of COL10A1 in BLCA. The relationship between COL10A1 and the immune cell infiltration, and immune checkpoint genes were evaluated. GO/KEGG enrichment analyses were used to analyze potential mechanisms between the high and low COL10A1 groups. COL10A1 protein expression levels of seventy-seven tumor tissue and five corresponding adjacent normal tissue from BLCA patients were analyzed by immunohistochemical staining, and high COL10A1 protein expression is associated with poor survival. Finally, we identified that COL10A1 is an unfavorable factor for BLCA, and its expression is significantly connected with the tumor-infiltrating immune cells.

## Materials and methods

### Acquisition of data

The mRNA sequencing data (FPKM format) for normal and primary tumor samples were downloaded from the TCGA database (https://portal.gdc.cancer.gov/, up to December 28, 2021), including 405 BLCA samples and 19 paired normal bladder samples. We also downloaded Gene Expression Omnibus (GEO) (https://www.ncbi.nlm.nih.gov/) and ArrayExpress database (https://www.ebi.ac.uk/arrayexpress/) for validation, including GEO datasets GSE13507 (28, 29), GSE31684 (30, 31), GSE32548 (32), GSE32894 (33) and, ArrayExpress datasets E-MTAB-4321 (34), E-MTAB-1803 (35). The BLCA patients’ clinical data from the TCGA database, GEO database, and ArrayExpress database have also been attained. Patients were subdivided into high and low groups according to the cutoff point of COL10A1 mRNA expression. A total of 1579 BLCA samples (TCGA:405; GSE13507:165; GSE31684:93; GSE32548:131; GSE32894:224; E-MTAB-4321:476; E-MTAB-1803:85) and 86 normal tissues (TCGA:19; GSE13507: 67) were included in this study.

### Expression profile of COL10A1 in BLCA

The expression of COL10A1 gene in BLCA is analyzed using gene expression profiling interactive analysis 2 database (GEPIA2, http://gepia2.cancer-pku.cn/), a web tool providing differential expression analysis, profiling plotting, correlation analysis, patient survival analysis, similar gene detection, and dimensionality reduction analysis based on TCGA and GTEx data (36). The mRNA levels of COL10A1 in different types of cancer were determined through analysis in the TIMER database (https://cistrome.shinyapps.io/timer/), a comprehensive web server for systematical analysis of immune infiltrates across diverse cancer types. COL10A1 mRNA expression in various aspects including, tumor tissues, normal tissues, age, sex, tumor grade, tumor stage, and molecular subtype of BLCA (Basal-squamous, Luminal, Luminal-infiltrated, Luminal-papillary, and Neuronal) in TCGA database, GEO database, and ArrayExpress database were also evaluated by using R language.

### Clinical specimens and immunohistochemistry staining

Seventy-seven BLCA tumor tissues, five adjacent normal tissues, and patients’ clinical data were obtained from BLCA patients undergoing surgical resection at West China hospital, China, from December 2009 to May 2012, fixed by formalin and embedded by paraffin. The study was conducted in accordance with the Declaration of Helsinki and the study was performed with the permission of the Biomedical Research Ethics Committee of West China Hospital of Sichuan University (2020366). All patients signed informed consent for the use of their information and samples for research.

Immunohistochemistry staining was performed to examine the protein expression of COL10A1 in BLCA tissues. Immunohistochemistry was conducted following the manufacturer’s instructions of the immunohistochemical secondary antibody kit (abs996, absin, Shanghai, China). The paraffin sections were dewaxed, rehydrated, placed in citrate buffer for antigen retrieval, and blocked in 3% H_2_O_2_. Then, the sections were incubated with primary COL10A1 antibody (1:250, mouse, ab49945, Abcam, USA) at room temperature for 30 minutes, followed by visualization with the DAB chromogen solution. Images were captured using a Zeiss microscope equipped with a digital camera. Staining was independently evaluated by two experienced pathologists blinded to patients’ clinical information. The score for COL10A1 staining was based on the proportion of immune-positive cells and the staining intensity. The proportion of immune-positive cells was scored as followings: 0: <5%; 1:6%- 25%; 2: 26%- 50%; 3: 51%- 75%; and 4: > 75%. Staining intensity was quantified as follows: 1: negative; 2: weak; 3: medium; and 4: strong. The staining score was calculated as the score of staining intensity × the score of the proportion of immune-positive cells.

### Enrichment analysis of COL10A1 gene co-expression network in BLCA

Firstly, we identified co-expressed genes associated with COL10A1 expression in the TCGA-BLCA datasets in R software and retained only protein-coding genes. We used Pearson’s correlation coefficient to test the statistical correlation and the ggplot2 package of R software to draw the volcano map and heat map for display. We conducted Gene Ontology (GO) function and Kyoto Encyclopedia of Genes and Genomes (KEGG) pathway enrichment analysis of co-expressed genes on the DAVID website (https://david.ncifcrf.gov/) (37) and enriched gene terms with FDR (False Positive Rate) q value <0.05 were considered statistically significant, results were visualized as bubble plots in R software.

### Gene set enrichment analysis

To investigate the potential regulatory mechanisms of COL10A1, we divided samples from the TCGA BLCA datasets into two groups according to the cutoff point of COL10A1 expression level and performed GSEA using the GSEA software (version 4.2.1) (www.gsea-msigdb.org/gsea/index.jsp) (38) with the annotated gene sets in “h.all.v7.5.symbols.gmt (Hallmarkers)” chosen as the reference gene sets to investigate whether genes in the two groups were rich in meaningful biological processes. FDR (qvalue) <0.05 were considered statistically significant.

### Tumor mutation burden analysis

Somatic mutations and somatic copy number alternations (CNAs) data of BLCA were downloaded from the TCGA database. The “maftools” R package was used to display the mutation details of the genes with the top 20 mutation frequencies between the high and low COL10A1 group in the waterfall plot. We compared the transcription levels of COL10A1 between wild and mutation groups of genes with the top 20 mutation frequencies.

### Tumor immune infiltrating analysis

Tumor Immune Estimation Resource (TIMER, http://timer.cistrome.org/) web server is a comprehensive resource for systematical analysis of immune infiltrates across diverse cancer types (39). The correlation between COL10A1 expression and the infiltration level of six types of immune cells (B cell, CD4+ and CD8+ T cell, M1 and M2 macrophage, eosinophil, neutrophil, monocyte, dendritic cells, natural killer cell: NK cell, general T cells, Follicular helper T cell: Tfh, tumor-associated macrophage: TAM, mast cell, T-helper 1 cell: Th1 cell, Th2 cell, Th17 cell and regulatory T cell: Treg) we assessed by using TIMER in BLCA. Comparison of tumor infiltration levels among tumors with different copy number variation (CNV) of COL10A1 using SCNA module on the TIMER website. We compared COL10A1 expression between six immune subtypes showed that distinct immune signatures based on the dominant sample characteristics of their tumor samples in the TCGA database (40). Fractions of 22 types of tumor-infiltrating immune cells were evaluated between the high and low COL10A1 group in BLCA by applying the CIBERSORT algorithm (41). Besides, gene expression correlation analysis between COL10A1 and immune marker of immune cells in the TCGA database was performed by using the spearman method to determine the correlation coefficient on the GEPIA2 web servers, in which normal tissue datasets were used as the control. The mRNA levels of 10 immune-checkpoint genes between the high and low COL10A1 group were also assessed.

### Statistical analysis

The statistics in this study were performed by using R language (Version 3.6.2), a language and environment for statistical computing (R foundation for statistical computing, Vienna, Austria, https://www.R-project.org/), and GraphPad Prism software (Version 8.0.2). Quantitative data are presented as the mean ± standard derivation. The chi-square (*x* ^2^) test was utilized to evaluate the correlation between COL10A1 expression and clinicopathological features of patients. The significance of the difference between groups was determined by the Student’s t-test (unpaired, two-tailed) and one-way analysis of variance (ANOVA). Logistic regression analysis was used to assess the correlations between the clinical characteristics and COL10A1 expression level. “Survminer” and “survival” R packages were utilized in R language to determine cutoff points and the survival difference of overall survival between the high and low COL10A1 group by Kaplan-Meier analysis with a log-rank test. *P*< 0.05 was considered statistically significant.

## Results

### COL10A1 expression is elevated in BLCA

**Figure 1:**
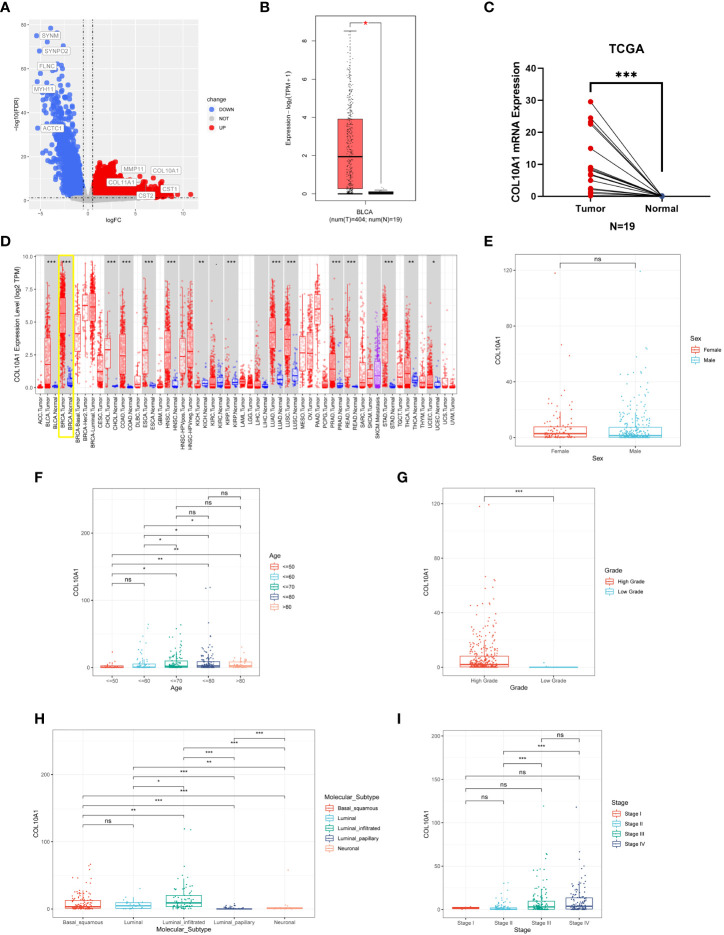
The expression of COL10A1 in BLCA and pan-carcinoma. **(A)** Differential genes in BLCA tissues compared with adjacent normal tissues based on the TCGA database. **(B)** The mRNA expression of COL10A1 in BLCA and matched non-carcinoma tissues based on the GEPIA database. **(C)** Paired expression analysis of COL10A1 in normal and BLCA tissues based on TCGA database. **(D)** COL10A1 expression data in various cancer types based on the TIMER database. To study the correlation of COL10A1 expression with clinical-pathological parameters, including sex **(E)**, age **(F)**, grade **(G)**, molecular subtypes **(H)** and stage **(I)** in BLCA patients. *P < 0.05; **P < 0.01; ***P < 0.001, ns, not significant.

The RNA-seq data from the TCGA database were used to compare COL10A1 expression between tumor samples and adjacent normal tissues using the R language and TIMER database. COL10A1 expression was markedly and significantly increased in BLCA (*P*<0.05, **Figures 1A–C**), breast invasive carcinoma (BRCA), cholangiocarcinoma (CHOL), colon adenocarcinoma (COAD), esophageal carcinoma (ESCA), head and neck squamous cell carcinoma (HNSC), lung adenocarcinoma (LUAD), lung squamous cell carcinoma (LUSC), prostate adenocarcinoma (PRAD), rectum adenocarcinoma (READ), stomach adenocarcinoma (STAD), thyroid carcinoma (THCA) and, uterine corpus endometrial carcinoma (UCEC) (*P*<0.05, **Figure 1D**). No significant difference in COL10A1 level was shown between male and female cases in the TCGA-BLCA database (*P*>0.05, **Figure 1E**). We evaluated the expression levels of COL10A1 in different age groups in the TCGA-BLCA database, where older patients have higher expression of COL10A1 (*P*<0.01, **Figure 1F**). COL10A1 mRNA level was observed to be higher in high grade than in low grade in the TCGA database (*P*<0.001, **Figure 1G**). The COL10A1 levels in mRNA expression-based molecular subtypes were further evaluated (*P*<0.05; **Figure 1H**) (42). Besides, COL10A1 was also upregulated in stage III and stage IV than stage II cases in the TCGA database (*P*<0.001, **Figure 1I**). The optimal cutoff value was used to create a categorical dependent variable based on COL10A1 expression. As shown in **Table 1**, COL10A1 mRNA expression level was significantly associated with tumor grade (High vs. Low, *P*<0.001) and pathological stage (III&IV vs. I&II, *P*< 0.001) in the TCGA database. We also compared COL10A1 expression levels among clinicopathological subgroups in the GSE13507, GSE31684, GSE32548, GSE32894, E-MTAB-4321, and E-MTAB-1803 datasets (**Supplementary Figures 1A–F**).

**Table 1:** Relationship between COL10A1 expression and clinicopathological parameters in BLCA.

Clinicopathological parameters	TCGA cohort (n=394)						Validation cohort (n=77)					
	COL10A1 expression						COL10A1 expression					
	Group	n	Mean	SD	*t*	*P*	Group	n	Mean	SD	*t*	*P*
Age	<80	225	6.65	12.21	-0.66	0.510	<=60	30	7.60	3.93	-1.48	0.145
	>80	169	7.61	15.69			>60	47	8.92	3.62		
Sex	Female	103	7.69	15.50	0.49	0.622	Female	6	5.67	2.73	-2.47	**0.044**
	Male	291	6.84	13.17			Male	71	8.63	3.77		
Grade	High	374	7.43	14.07	9.64	**0.000**	High	54	9.43	3.67	4.40	**0.000**
	Low	20	0.23	0.74			Low	23	6.00	2.86		
Stage	I-II	127	2.26	5.10	-6.61	**0.000**	I-II	49	6.76	2.94	-5.97	**0.000**
	III-IV	267	9.35	15.90			III-IV	28	11.29	3.34		

Bold values indicate *P* < 0.05.

### High expression of COL10A1 indicates poor prognosis of BLCA patients in BLCA datasets

**Figure 2:**
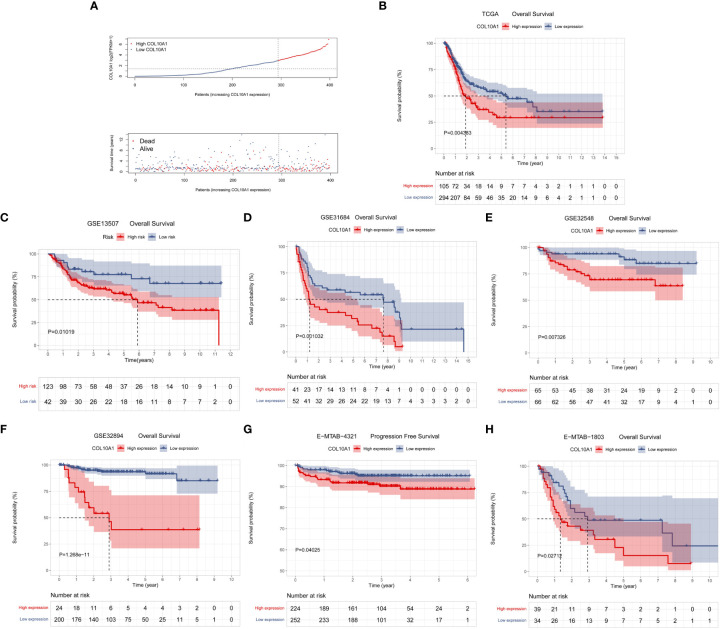
The COL10A1 expression level was associated with the prognosis of BLCA patients. **(A)** The distribution of COL10A1 expression and survival status of BLCA patients in the TCGA cohort. Kaplan–Meier analyses to evaluate the correlation between COL10A1 expression and the overall survival (OS) of BLCA patients in the TCGA **(B)**, GSE13507 **(C)**, GSE31684 **(D)**, GSE32548 **(E)**, GSE32894 **(F)**, E-MTAB-1803 **(H)** cohorts and progression free survival (PFS) in the E-MTAB-4321 **(G)** cohort indicated that higher COL10A1 expression was correlated to poorer prognosis of BLCA patients.

To explore the prognostic value of COL10A1 in the TCGA-BLCA database, patients were divided into high and low BLCA expression groups based on the optimal cutoff calculated *via* “survival” and “survminer” packages (**Supplementary Figure 2A**). The distribution of COL10A1 expression and survival status of BLCA patients were displayed in **Figure 2A**. We next evaluated the prognostic significance of COL10A1 expression in the TCGA-BLCA datasets using Kaplan-Meier analysis. Kaplan-Meier survival curves were generated based on the cutoff point of COL10A1 expression in BLCA and demonstrated that BLCA patients with high COL10A1 expression levels showed poor OS rate (*P*<0.01, **Figure 2B**).

### Validation of the elevated expression of COL10A1 in external datasets and biospecimens

To validate the relationship between COL10A1 expression and poor prognosis of BLCA, we performed gene expression analysis in subgroups and Kaplan-Meier analysis in the GEO database and ArrayExpress database based on the optimal cutoff points (**Supplementary Figures 2B–G**). In the GSE13507, GSE31684, GSE32548, GSE32894, E-MTAB-4321, and E-MTAB-1803 datasets, the results revealed that high COL10A1 expression was positively correlated with poor survival outcomes (*P*<0.05, **Figures 2C–H**).

**Figure 3:**
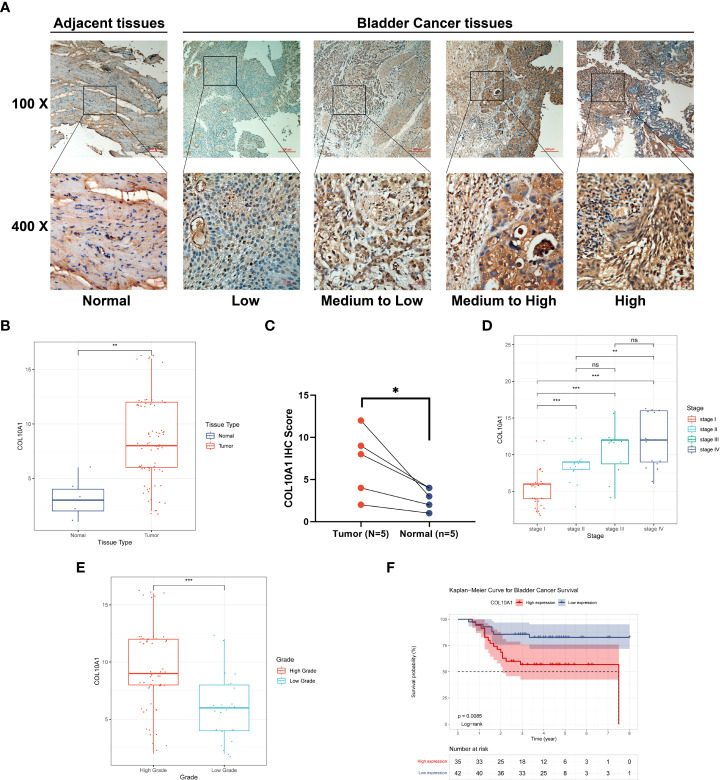
Validation of the increased expression of COL10A1 and prognostic value in 77-patients cohort. **(A)** Representative IHC staining for COL10A1 in BLCA and normal tissues. Protein expression of COL10A1 based on IHC staining scores was significantly different in BLCA and adjacent normal tissues **(B, C)**, also in tumor grade subgroup **(D)**, and stage subgroup **(E)**. Kaplan–Meier plot verified that high COL10A1 protein levels were associated with poorer survival outcomes in 77 BLCA patients **(F)**. *P < 0.05; **P < 0.01; ***P < 0.001. ns, not significant.

Eighty-two human clinical samples, including seventy-seven BLCA tissues and five corresponding adjacent normal tissues, were collected. IHC was conducted to evaluate the COL10A1 protein expression in BLCA and corresponding adjacent normal tissues (**Figure 3A**). Protein expression analysis showed that COL10A1 protein was significantly high-expressed in tumor tissues compared with adjacent normal tissues (*P*<0.001, **Figures 3B, C**). Further analysis demonstrated that the COL10A1 protein was strikingly correlated to the pathological stage (*P*< 0.05, **Figure 3D**) and tumor grade (*P*< 0.05, **Figure 3E**). COL10A1 protein level was also significantly associated with sex (male vs. female, *P*= 0.044), tumor grade (High vs. Low, *P*< 0.001), and pathological stage (III&IV vs. I&II, *P*< 0.001) in the validation cohort (**Table 1**, n=77). Furthermore, we explored the prognostic value of COL10A1 protein expression in BLCA. The median histochemical score was set as the cutoff value in survival analysis. Kaplan–Meier curve revealed that BLCA patients with high COL10A1 protein have shorter OS than those with lower COL10A1 protein (*P*= 0.0085, **Figure 3F**).

### Enrichment analysis of COL10A1 gene co-expression network in BLCA

**Figure 4:**
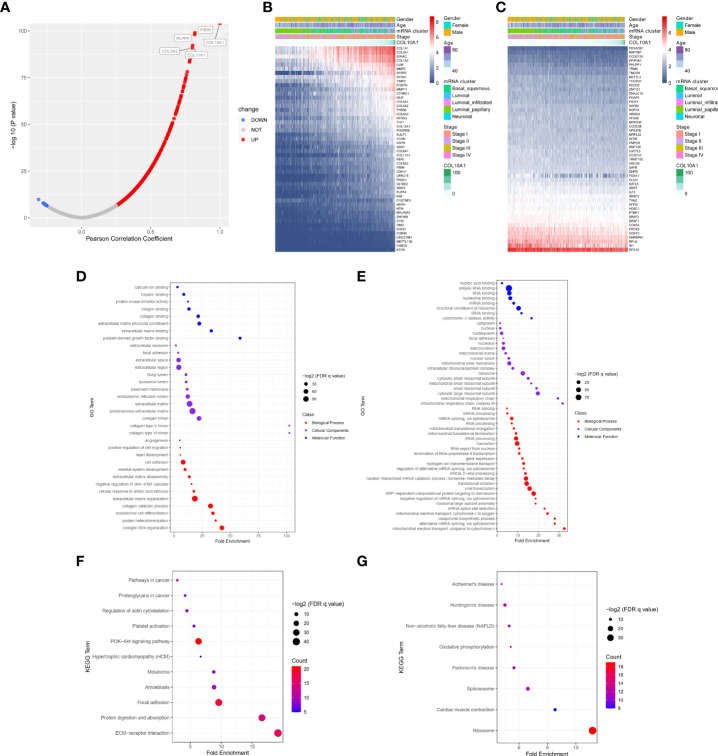
Enrichment analysis of COL10A1 gene co-expression network in BLCA. **(A)** The volcano map showed co-expression genes associated with COL10A1 expression in the TCGA-BLCA datasets. **(B, C)** Heat maps showed the top 50 co-expression genes positively and negatively correlated with COL10A1 expression in the TCGA-BLCA datasets. **(D, F)** Enrichment analysis of gene ontology (GO) terms and Kyoto Encyclopedia of Genes and Genomes (KEGG) terms for co-expression genes positively correlated with COL10A1. **(E, G)** Enrichment analysis of GO terms and KEGG terms for co-expression genes negatively correlated with COL10A1.

As shown in the volcano plot (**Figure 4A**), 2696 genes were positively correlated with COL10A1 expression level, and 4654 genes were significantly negatively correlated with expression level (*P*< 0.05) in the TCGA-BLCA data. The heat map (**Figures 4B, C**) showed that the top 50 genes positively and negatively correlated with COL10A1 expression, respectively. The detailed description of co-expressed genes is shown in **Supplementary Table 1**.

The genes with the strongest associations were FIBIN (cor= 0.820, *P*= 1.02E-99), PLPP4 (cor= 0.818, *P*=9.32E-99), COL11A1 (cor= 0.804, *P*= 6.98E-93) and, COL5A2 (cor = 0.801, *P*= 1.23E-91), when cor> 0.8 and *P*< 0.05 were set as the cutoff values.

GO and KEGG analyses were conducted to evaluate the top 200 co-expressed genes positively and negatively correlated with COL10A1 expression level *via* the R software package and DAVID website under FDR<0.05. We discovered that co-expression of COL10A1 was positively associated with multiple biological processes, including extracellular matrix, protein modification, and molecular binding (**Figure 4D**), and negatively correlated with RNA binding, RNA processing, RNA splicing, and biological process of mitochondrion (**Figure 4E**) in GO analysis.

The KEGG pathway enrichment analysis demonstrated that the top 200 co-expressed genes positively correlated with COL10A1 expression level were primarily involved in ECM-receptor interaction, protein digestion, and absorption, focal adhesion, and PI3K-Akt signaling pathway (**Figure 4F**). The bubble plot also revealed that KEGG terms, such as ribosome, spliceosome, and Huntington’s disease enriched in the co-expression group negatively correlated with COL10A1 (**Figure 4G**). **Supplementary Table 2** summarized the details of the GO and KEGG enrichment analyses of COL10A1 co-expression in the TCGA database.

### Gene set enrichment analysis

**Figure 5:**
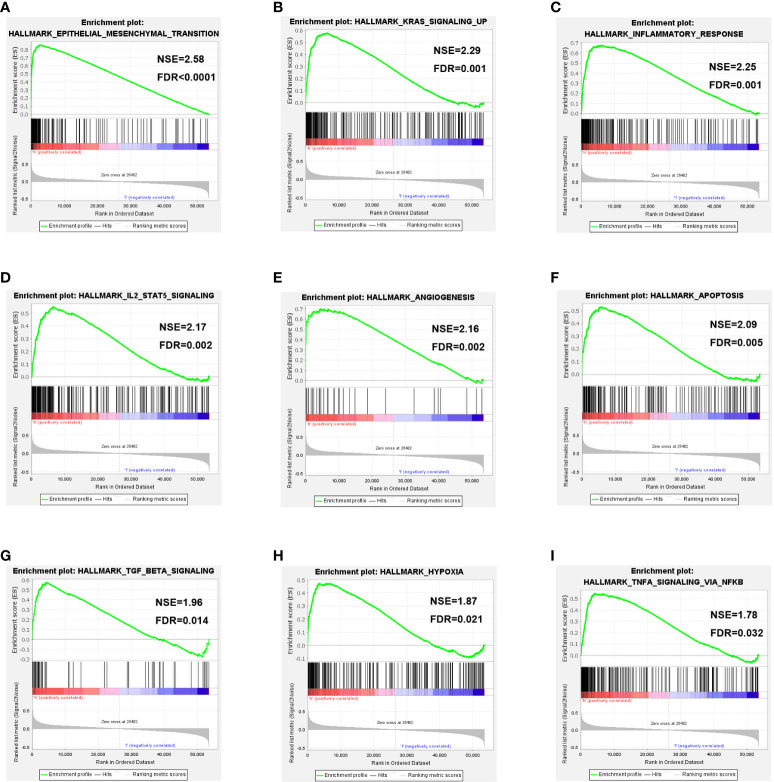
Gene set enrichment analysis. Pathway enriched in the epithelial-mesenchymal transition (EMT, **A**), KRAS signaling up **(B)**, inflammatory response **(C)**, IL2-STAT5 signaling **(D)**, angiogenesis **(E)**, apoptosis **(F)**, TGF-β signaling **(G)**, hypoxia **(H)** and TNF-α signaling *via* NF-κB **(I)** pathways.

To further investigate the potential function of COL10A1 in BLCA, GSEA analysis was conducted based on COL10A1 level in the TCGA database. The GSEA showed that substantial gene sets were positively enriched in COL10A1 high-expression group including epithelial-mesenchymal transition (EMT, NES= 2.58, FDR< 0.0001, **Figure 5A**), KRAS signaling up (NES= 2.29, FDR= 0.001, **Figure 5B**), inflammatory response (NES= 2.25, FDR= 0.001, **Figure 5C**), IL2-STAT5 signaling (NES= 2.17, FDR= 0.002, **Figure 5D**), angiogenesis (NES= 2.16, FDR= 0.002, **Figure 5E**), apoptosis (NES= 2.09, FDR= 0.005, **Figure 5F**), TGF-β signaling (NES= 1.96, FDR= 0.014, **Figure 5G**), hypoxia (NES= 1.87, FDR= 0.021, **Figure 5H**) and TNF-α signaling *via* NF-κB (NES= 1.78, FDR= 0.032, **Figure 5I**) pathways. Detailed GSEA analysis information is displayed in **Supplementary Table 3**.

### COL10A1 expression levels are associated with tumor mutational burden

**Figure 6:**
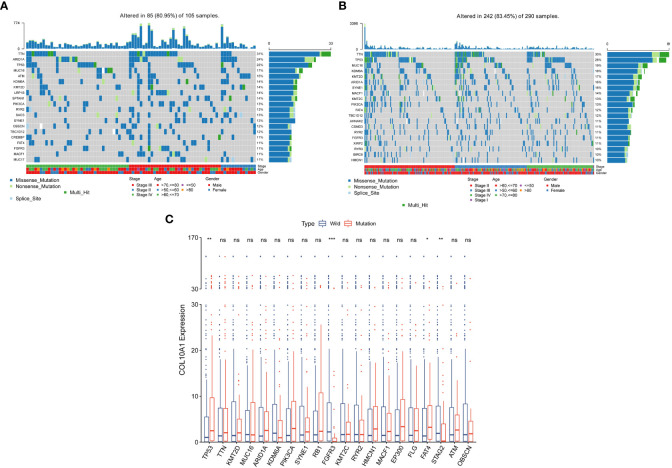
Somatic mutation analysis in high and low expression groups of COL10A1. Waterfall plot of the top 20 mutational genes in the high COL10A1 group **(A)** and low COL10A1 group **(B)**. Transcriptional levels of COL10A1 between wild and mutational types of top 20 genes with the highest mutation frequencies **(C)**. *P < 0.05; **P < 0.01; ***P < 0.001. ns, not significant.

To determine whether COL10A1 expression levels were associated with specific genomic characteristics in BLCA, we performed somatic mutation analysis based on COL10A1 expression levels by using the “maftools” package in the TCGA-BLCA database, in which the top 20 mutational genes were displayed. A high frequency of mutations in TTN (31%), ARID1A (24%), TP53 (22%), MUC16 (17%), and ATM (16%) in the high COL10A1 group (**Figure 6A**), whereas TTN (30%), TP53 (28%), MUC16 (19%), KDM6A (17%), and KMT2D (16%) were more frequently mutated in the low COL10A1 group (**Figure 6B**). The summary information of mutation data is shown in **Supplementary Figures 3A, B**.

Transcriptional levels of COL10A1 between wild and mutational types of the top 20 genes with the highest mutation frequencies were analyzed. Notably, the result suggested a higher somatic mutation burden of TP53 and FAT4 in the high COL10A1 group than in the low COL10A1 group. In contrast, the somatic mutation burden associated with FGFR3 and STAG2 was higher in the low COL10A1 group than in the high COL10A1 group (**Figure 6C**).

### Relationship between COL10A1 and immune cells infiltration

**Figure 7:**
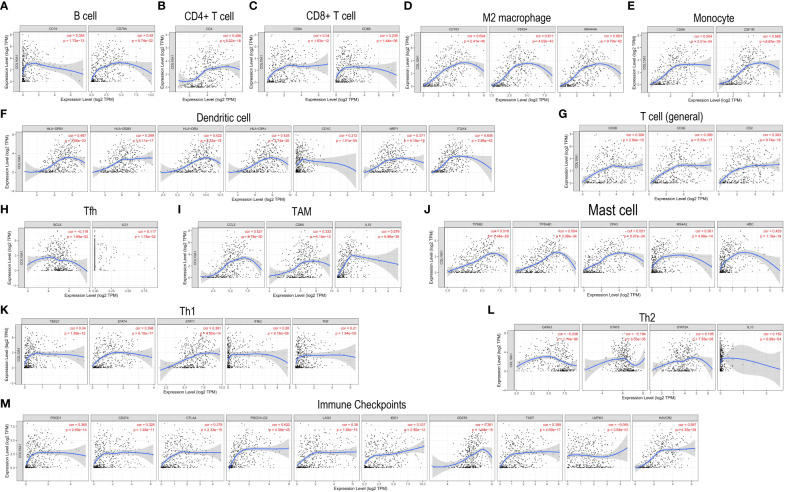
COL10A1 expression is related to immune infiltration degrees in BLCA. Spearman correlations between COL10A1 expression and twelve infiltrating immunocyte abundances: B cell **(A)**, CD4+ T cell **(B)**, CD8+ T cell **(C)**, M2 macrophage **(D)**, monocyte **(E)**, dendritic cells **(F)**, general T cells **(G)**, Tfh cell **(H)**, TAM **(I)**, mast cell **(J)**, Th1 cell **(K)**, Th2 cell **(L)**, and immune checkpoints genes **(M)**.

**Figure 8:**
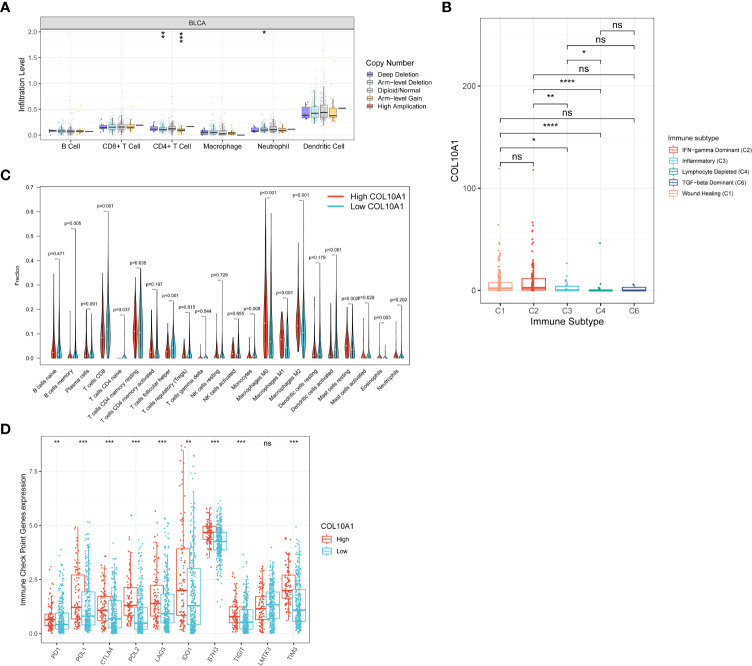
Correlation between COL10A1 and tumor immune infiltrating cells. COL10A1 CNV affects the infiltrating levels of CD4+ T cells in BLCA **(A)**. COL10A1 expression varied among different immune subtypes **(B)**. Changes of 22 immune cell subtypes between high and low COL10A1 expression groups in BLCA tumor samples **(C)**. Immune checkpoint genes expression between patients with high and low COL10A1 levels **(D)**. *P < 0.05; **P < 0.01; ***P < 0.001; ****P < 0.0001. ns, not significant.

To determine whether COL10A1 expression was related to immune cell infiltration in BLCA, we utilized the “Gene” module of the TIMER website to approximately study the correlations. As shown in **Figures 7A–M**, COL10A1 expression level exhibited a significant correlation with the levels of twelve immune cells assessed (B cell, CD4+ and CD8+ T cell, M2 macrophage, monocyte, dendritic cells, general T cells, Tfh cell, TAM, mast cell, Th1 cell and Th2 cell) and immune checkpoints (*P*<0.05), and not significantly associated with the expression level of eosinophils, neutrophils, NK cell, Th17, and Treg. Tumor infiltration levels differed with different CNV of COL10A1, infiltration levels of CD4+ T cell (*P*<0.01) were lower with chromosome arm-level deletion and gain of COL10A1 (**Figure 8A**). Additionally, COL10A1 expression varied among different immune subtypes, which was highest in the IFN-γ dominant subtype and was lowest in the lymphocyte- depleted subtype (**Figure 8B**).

To deeply confirm the role of COL10A1 in the tumor immune microenvironment, we took advantage of the CIBERSORT algorithm to evaluate the levels of 22 types of immune cells. BLCA samples in the TCGA database were assigned to a high or low COL10A1 expression group based on the optimal cutoff point. The fractions of M0 macrophages, M1 macrophages, M2 macrophages, resting mast cells, and eosinophils were distinctly increased in samples with high COL10A1 expression. However, memory B cells, CD8^+^ T cells, naive CD4^+^ T cells, Tfh cells, monocytes, activated dendritic cells, and activated mast cells in samples with high COL10A1 expression decreased (*P*< 0.05, **Figure 8C**). Furthermore, we analyzed the correlation between the expression level of COL10A1 and gene markers of immune cells, including B cell, T cell (general), CD4+ and CD8+ T cell, monocyte, mast cell, TAM, M1, and M2 macrophage, neutrophil, NK cell, Dendritic cell, Th1, Th2, Tfh, Th17, Treg and immune checkpoints in BLCA, using normal tissues as the control (**Table 2**).

**Table 2:** Correlation analysis between COL10A1 and immune cell marker and immune checkpoint genes in GEPIA.

Immune cell types	Gene	BLCA			
		Tumor		Normal	
		Correlation	*P* value	Correlation	*P* value
B cell	CD19	0.38	**2.90E-15**	-0.18	4.70E-01
	CD79A	0.46	**1.60E-22**	-0.05	8.40E-01
T cell (general)	CD3D	0.30	**1.10E-09**	0.02	9.50E-01
	CD3E	0.41	**3.60E-18**	0.00	9.90E-01
	CD2	0.40	**4.70E-17**	0.19	**4.00E-02**
CD4+ T cell	CD4	0.43	**8.70E-20**	0.36	1.30E-01
CD8+ T cell	CD8A	0.36	**1.00E-13**	0.27	2.60E-01
	CD8B	0.25	**5.50E-07**	0.15	5.30E-01
Monocyte	CD86	0.56	**3.10E-34**	0.20	4.20E-01
	CSF1R	0.60	**6.40E-41**	0.24	3.30E-01
Mast cell	TPSB2	0.50	**1.70E-26**	0.35	1.40E-01
	TPSAB1	0.57	**9.60E-36**	0.33	1.60E-01
	CPA3	0.57	**8.30E-37**	0.55	**1.40E-02**
	MS4A2	0.39	**1.80E-16**	0.46	**4.60E-02**
	HDC	0.47	**2.30E-23**	0.39	9.70E-02
TAM	CCL2	0.54	**2.40E-31**	0.48	**3.80E-02**
	CD68	0.36	**7.50E-14**	-0.01	9.80E-01
	IL10	0.57	**2.50E-36**	0.12	6.30E-01
M1 Macrophage	NOS2	0.13	**6.60E-03**	0.01	9.80E-01
	IRF5	0.06	2.00E-01	-0.13	5.90E-01
	PTGS2	0.22	**9.40E-06**	0.41	7.90E-02
M2 Macrophage	CD163	0.63	**1.90E-45**	0.11	6.60E-01
	VSIG4	0.62	**4.70E-44**	0.29	2.30E-01
	MS4A4A	0.61	**4.20E-43**	0.33	1.70E-01
Neutrophils	CEACAM8	0.01	8.10E-01	-0.48	**3.70E-02**
	ITGAM	0.58	**6.60E-38**	0.23	3.50E-01
	CCR7	0.12	**1.80E-02**	-0.12	6.40E-01
NK cell	KIR2DL1	0.27	**5.60E-08**	-0.12	4.10E-01
	KIR2DL3	0.22	**8.50E-06**	-0.24	3.20E-01
	KIR2DL4	0.26	**1.10E-07**	-0.11	6.60E-01
	KIR3DL1	0.24	**9.00E-07**	-0.02	8.10E-01
	KIR3DL2	0.21	**3.30E-05**	0.19	**4.40E-02**
	KIR3DL3	0.06	2.10E-01	-0.21	**2.80E-02**
	KIR2DS4	0.16	**1.50E-03**	-0.16	9.70E-02
Dendritic cell	HLA-DPB1	0.48	**2.80E-24**	0.24	**1.20E-02**
	HLA-DQB1	0.35	**9.20E-13**	0.29	**2.30E-03**
	HLA-DRA	0.44	**2.70E-20**	0.10	6.90E-01
	HLA-DPA1	0.45	**3.10E-21**	0.09	7.00E-01
	CD1C	0.22	**5.20E-06**	-0.04	8.80E-01
	NRP1	0.41	**1.30E-17**	0.64	**3.20E-03**
	ITGAX	0.60	**1.50E-40**	0.21	3.80E-01
Th1	TBX21	0.35	**4.20E-13**	0.09	7.10E-01
	STAT4	0.42	**6.90E-19**	0.10	6.70E-01
	STAT1	0.39	**7.70E-16**	0.61	**6.00E-03**
	IFNG	0.31	**2.10E-10**	0.06	8.00E-01
	TNF	0.23	**2.00E-06**	-0.01	9.80E-01
Th2	GATA3	-0.24	**1.50E-06**	0.01	9.60E-01
	STAT6	-0.15	**1.80E-03**	0.32	1.80E-01
	STAT5A	0.21	**2.60E-05**	0.25	3.00E-01
	IL13	0.18	**4.00E-04**	0.13	6.00E-01
Tfh	BCL6	-0.08	1.30E-01	0.24	3.20E-01
	IL21	0.15	**2.40E-03**	-0.06	8.00E-01
Th17	STAT3	0.32	**3.30E-11**	0.42	7.50E-02
	IL17A	-0.05	3.20E-01	-0.10	7.00E-01
Treg	FOXP3	0.47	**2.40E-23**	-0.10	6.90E-01
	CCR8	0.48	**7.10E-25**	0.06	8.20E-01
	STAT5B	0.08	1.00E-01	0.46	**4.80E-02**
	TGFB1	0.30	**7.30E-10**	0.33	1.70E-01
Immune Checkpoints	PDCD1	0.38	**1.50E-15**	0.19	4.40E-01
	CD274/PD-L1	0.34	**1.10E-12**	0.03	8.90E-01
	CTLA4	0.39	**1.80E-16**	0.11	6.50E-01
	PDCD1LG2/PD-L2	0.64	**3.80E-47**	0.35	1.40E-01
	LAG3	0.38	**5.50E-15**	0.26	2.80E-01
	IDO1	0.35	**2.10E-13**	-0.10	6.70E-01
	CD276/B7H3	0.39	**3.80E-16**	0.41	8.20E-02
	TIGIT	0.40	**4.20E-17**	0.06	8.20E-01
	LMTK3	-0.03	5.10E-01	-0.30	2.10E-01
	HAVCR2	0.59	**6.90E-40**	0.22	3.70E-01

Bold values indicate *P* < 0.05.

Finally, immune checkpoint gene expressions between patients with high and low COL10A1 levels were evaluated in the TCGA database. Results demonstrated that nine genes (PD1, PDL1, CTLA4, PDL2, LAG3, IDO1, B7H3, TIGIT, TIM3) were up-expressed in the high COL10A1 group, which indicates COL10A1 might associate with immune response in BLCA (**Figure 8D**).

## Discussion

Bladder cancer is one of the deadly urinary malignancies, and the prognosis is still very poor. At present, traditional treatment options also have certain limitations in improving the survival outcome of patients, including surgery and chemotherapy (43, 44). Meanwhile, immune checkpoint inhibitor therapeutics provide patients with better surveillance opportunities, unique treatment options, and greater hope of prolonged survival (5). Therefore, finding new biomarkers associated with the immunomodulation of BLCA is critical to its diagnosis, treatment, and prognosis.

Collagen is the main component of the extracellular matrix, and more and more studies have confirmed that collagen can promote tumorigenesis and metastasis (45). In recent years, it has been found that collagen can play an immunomodulatory role in the tumor microenvironment, especially in tumor-related macrophages and T cells (46, 47), thus affecting tumor progression, prognosis, and immunotherapy response (47). The immunomodulatory effects of tumor-associated collagen may provide a basis for the development of current therapeutic strategies and new therapeutic approaches for tumors (48). The alpha chain of type X collagen encoded by the COL10A1 gene belongs to the collagen family, a short-chain collagen expressed by hypertrophic chondrocytes during endochondral ossification (15). COL10A1 has not been fully studied, but can serve as a potential molecular marker for a wide variety of tumors, including BLCA.

Pan-cancer characterization of expression based on the TCGA database showed that COL10A1 was significantly overexpressed in 13 cancer types than in normal tissues, (**Figure 1C**). However, COL10A1 was low expressed in 2 tumor types, comprising KICH and KIRP, which might be due to the diverse tumorigenic mechanisms. In this study, we used the IHC method to detect the protein expression level of COL10A1 in seventy-seven BLCA tissues and five adjacent tissues samples, and the results were consistent with the above bioinformatics study (**Figure 3A**). In our study, high COL10A1 expression is associated with malignant clinicopathologic features like stage and grade. Then, we utilized clinical information from the TCGA database to evaluate the prognostic value of COL10A1 in BLCA and found that high expression of COL10A1 was significantly correlated with OS prognosis in BLCA patients (**Figure 2B**), which was validated in GEO and ArrayExpress databases (**Figures 2B–G**). In our cohort, we divided BLCA patients into two groups with high and low COL10A1 protein expression by IHC staining, and the Kaplan-Meier survival curves indicated that high COL10A1 expression was likely to present a poor clinical outcome than those with low COL10A1 expression (**Figure 3F**), which further verified the analysis results of sequencing data. Collectively, the findings of this study indicate that COL10A1 is a promising diagnostic and prognostic biomarker in BLCA patients.

To uncover the mechanism hidden behind its invasive growth pattern, we constructed the COL10A1 gene co-expression network in the TCGA-BLCA datasets and performed GO and KEGG enrichment analyses. In the present study, the expressions of FIBIN, PLPP4, COL11A1, and COL5A2 in BLCA had the strongest correlation with COL10A1. PLPP4 (phospholipid phosphatase 4) could promote proliferation and tumorigenesis in lung carcinoma cells, and serve as a potential therapeutic target for glioma and PAAD (49, 50). COL11A1 is associated with poor clinical outcomes in numerous solid cancers and is a novel biomarker and a pivotal target in cancer (51). A retrospective analysis based on GSE13507 data showed that COL5A2 was correlated with poor survival outcomes (52). COL5A2 has been reported to be suitable for clinical prognostic prediction for MIBC patients (53). While the role of FIBIN in cancer has not been reported. GO and KEGG enrichment analyses based on co-expression are associated with many classical signaling pathways, such as the extracellular matrix, PI3K-Akt signaling, and ECM-receptor interaction. The GSEA analysis revealed that the differential genes grouped based on COL10A1 expression were mainly enriched in EMT, KRAS signaling up, inflammatory response, IL2-STAT5 signaling, angiogenesis, apoptosis, TGF-β signaling, hypoxia, and TNF-α signaling *via* NF-κB. Previous studies have elucidated the mechanistic link between COL10A1 and PI3K-Akt signaling pathway, EMT, inflammatory response, apoptosis, TGF-β signaling, and hypoxia in the occurrence and progression of BLCA (54–59). This study is the first to disclose the underlying correlation between COL10A1 and KRAS signaling up, IL2-STAT5 signaling, and TNF-α signaling *via* NF-κB in BLCA.

TMB can reflect the quantity of mutations in tumors and generate immunogenic neoantigens, which improves the possibility of T cell recognition, and clinically relates to better immune checkpoint inhibitors (ICIs) response (60). TMB, consistent with PD-L1 expression, could provide a reference for tumor patients to select ICIs treatment (61). In the present study, somatic mutation analysis based on COL10A1 expression levels was conducted in the TCGA database. We listed the top 20 genes with the highest mutation rates in the high and low COL10A1 groups (**Figures 6A, B**). TTN, TP53, MUC16, ARID1A, KMT2D, and KDM6A were the genes with the highest mutation frequencies in both groups, whereas ATM appeared in the top 5 of the high COL10A1 group. Loss-of-function mutations of ATM are a universal event in various malignancies, and genetic inactivation of ATM was shown to increase the sensitivity of tumors to radiotherapy (62). In addition, the boxplot of the correlation between COL10A1 expression level and gene mutation showed that the COL10A1 expression in the mutation type of TP53 and FAT4 were significantly higher than those in the wild types. In comparison, the COL10A1 expression with mutational FGFR3 and STAG2 were lower than those in the wild type (**Figure 6C**). TP53 is one of the most mutated genes in human cancers (63). The high mutation burden of TP53 is a potential target for cancer gene therapy (64). A population-based study in the United States revealed that TP53 mutations might predict outcomes in BLCA patients and are associated with more invasive disease, with a higher prevalence among hair dye users and individuals with higher arsenic exposure (65). Studies have shown that BLCA patients with TP53 mutation have a poor prognosis of OS (66, 67). FAT4 is a cadherin-related gene and is considered a tumor suppressor in multiple human cancers (68–70). However, no studies on the role of FAT4 in BLCA have been published. FGFR3 is one of the most frequently mutated genes and a noteworthy target in BC (71). Oncogenic FGFR3 mutations in BLCA were associated with a favorable prognosis and would be more likely to benefit from anti-FGFR3 therapy (72). STAG2 is one of four components of the cohesion complex and is frequently mutated in BLCA, which is related to an unfavorable prognosis. In summary, the mutation status of TP53, FAT4, FGFR3, and STAG2 is significantly correlated with the expression level of COL10A1, which will provide clues for in-depth mechanism research and targeted therapy development.

Immune-infiltrating cells, an important component of the tumor microenvironment, play an important role in influencing tumor growth, progression, therapeutic effect, and patient prognosis (73, 74). Higher immune infiltration in MIBC is associated with improved disease-specific survival (DSS) after bladder-sparing trimodality therapy (75). Studies have shown that higher RNA-based immune signature scores were significantly associated with complete pathological response (CR) and better progression-free survival (PFS) outcomes after pembrolizumab therapy (76). TIMER was used to explore the correlation of COL10A1 expression with immune cell infiltration levels in tumors, which showed that samples with high COL10A1 expression tended to harbor more B cells, CD4+ and CD8+ T cells, M2 macrophages, monocytes, dendritic cells, general T cells, Tfh cells, TAM, mast cells, Th1cells and Th2 cells) and fewer eosinophils, M1 macrophages, neutrophils, NK cells, Th17 cells, and Treg cells. Further, COL10A1 CNV was significantly correlated with the infiltration levels of CD4+ T cells and neutrophils. Besides, COL10A1 expression was highest in the IFN-γ dominant subtype, which had the highest M1/M2 macrophage polarization, a strong CD8 signal, the most remarkable T cell receptor diversity, and a high proliferation rate (40). These analyses showed that COL10A1 was involved in regulating the immunity of the tumor microenvironment in BLCA, especially in CD4+T cells, CD8+T cells, and M2 macrophages. In the analysis of infiltration levels of 22 kinds of immune cells in high and low COL10A1 expression groups by using the CIBERSORT algorithm, we also observed increased infiltration levels of M0 macrophages, M1 macrophages, M2 macrophages, resting mast cells, and eosinophils in high COL10A1 group, and decreased infiltration level of memory B cells, CD8^+^ T cells, naive CD4+ T cells, Tfh cells, monocytes, activated dendritic cells and, activated mast cells in high COL10A1 group. Through the analysis of the GEPIA web server, if we set the threshold of the correlation coefficient as 0.5 and *P*< 0.05 was considered statistically significant, we found that the expression of COL10A1 was significantly positively related to the gene markers of monocytes and M2 macrophages, suggesting that COL10A1 may affect the immune infiltration of BLCA by affecting the expression of monocytes and M2 macrophages. In summary, M2 macrophages may be the key points of COL10A1 expression affecting the immune microenvironment of BLCA. Macrophages are ubiquitous cellular components in all tissues and body compartments (77). Macrophages act as double-edged swords in cancer by exerting pro- and anti-tumor capabilities (78). M2-polarized macrophages are contributors to play a role in pro-tumor and anti-inflammation activity (79), which may be the underlying reason for the poor prognosis in BLCA patients with high COL10A1 expression. We speculate that COL10A1 may have an essential role in recruiting infiltrating immune cells and regulating immunity in BLCA, thus affecting prognosis. However, more research is needed to confirm this hypothesis, especially the effect of COL10A1 on the M2 polarization of macrophages in the BLCAmicroenvironment.

As an important component of the extracellular matrix, COL10A1 will play an important role in the diagnosis and development of new therapies for tumors. It is worth noting that liquid biopsy is an important part of the research and development of urinary tumor diagnostic technology (80, 81). Collagen, on the other hand, has the potential to become a research direction for liquid biopsy of tumors.

## Conclusion

In summary, our study demonstrated that COL10A1 is overexpressed in BLCA tissues and was associated with multiple clinicopathological features, verified by the IHC method in our cohort. Furthermore, the TCGA cohort, four GEO cohorts, two ArrayExpress cohorts, and our 77-patient cohort have all verified that high COL10A1 expression is significantly associated with poor prognosis of BLCA. Regarding biological functions, we demonstrated that COL10A1 was involved in EMT, KRAS signaling up, inflammatory response, IL2-STAT5 signaling, angiogenesis, apoptosis, TGF-β signaling, hypoxia, and TNF-α signaling *via* NF-κB in BLCA. Besides, COL10A1 expression is related to tumor mutational genes and filtration levels of various immune cells in tumor microenvironments. Taken together, these results suggest a latent role of COL10A1 as a prognostic marker and therapeutic target for BLCA in the future.

## Data availability statement

The datasets presented in this study can be found in online repositories. The names of the repository/repositories and accession number(s) can be found in the article/**Supplementary Material**.

## Ethics statement

The studies involving human participants were reviewed and approved by The Biomedical Research Ethics Committee of West China Hospital of Sichuan University. The patients/participants provided their written informed consent to participate in this study.

## Author contributions

PH was the sponsor of the study. XMW, FZ, YB, KC, DL, and RW assisted in collecting BLCA tissue samples and the clinical data. XMW was responsible for collecting and analyzing public data, completing experiments, drawing charts, and writing manuscripts. XW and YT reviewed and revised the article. All authors contributed to the article and approved the submitted version.

## Supplementary material

Supplementary Figure 1 The correlation of COL10A1 expression with clinical pathological parameters in BLCA patients in GSE13507 **(A)**, GSE31684 **(B)**, GSE32548 **(C)**, GSE62894 **(D)**, E-MTAB-4321 **(E),** and E-MTAB-1803 **(F)** datasets. **P* < 0.05; ***P* < 0.01; ****P* < 0.001. ns, not significant.

Supplementary Figure 2 The optimal cutoff value was used to create a categorical dependent variable based on COL10A1 expression in TCGA **(A)**, GSE13507 **(B)**, GSE31684 **(C)**, GSE32548 **(D)**, GSE62894 **(E)**, E-MTAB-4321 **(F),** and E-MTAB-1803 **(G)** datasets.

Supplementary Figure 3 The summary information of mutation data in the high **(A)** and low **(B)** COL10A1 groups in the TCGA-BLCA dataset.
