# Supplementary figures and images for: Prognostic value of COL10A1 and its correlation with tumor-infiltrating immune cells in urothelial bladder cancer: A comprehensive study based on bioinformatics and clinical analysis validation
Source: Front Immunol. 2023 Mar 17;14:955949. doi: 10.3389/fimmu.2023.955949 (PMC10063846; doi:10.3389/fimmu.2023.955949)

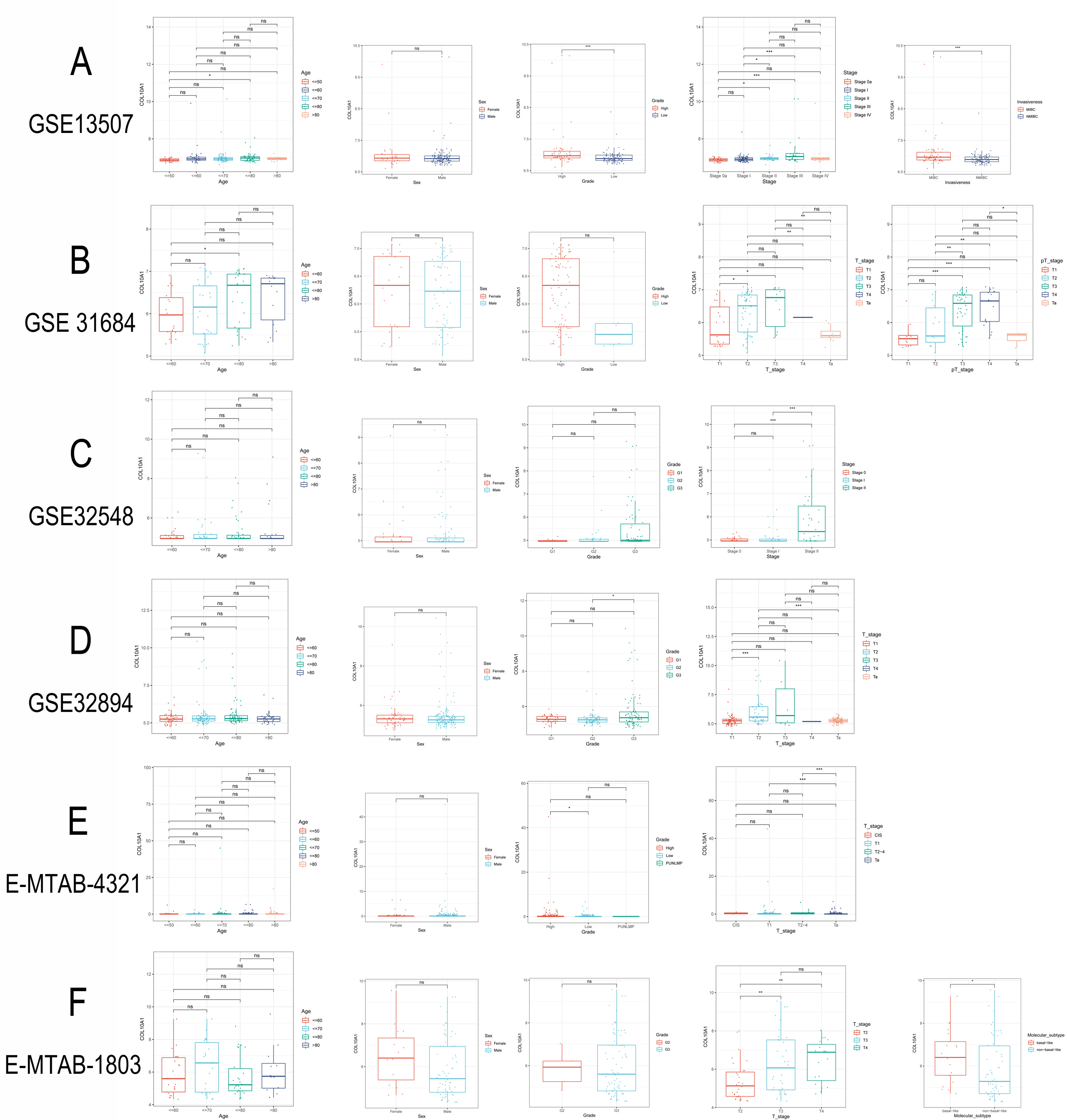

Supplement: Supplementary Figure 1 — The correlation of COL10A1 expression with clinical pathological parameters in BLCA patients in GSE13507 (A), GSE31684 (B), GSE32548 (C), GSE62894 (D), E-MTAB-4321 (E), and E-MTAB-1803 (F) datasets. *P < 0.05; **P < 0.01; ***P < 0.001. ns, not significant. [file Image_1.tif]

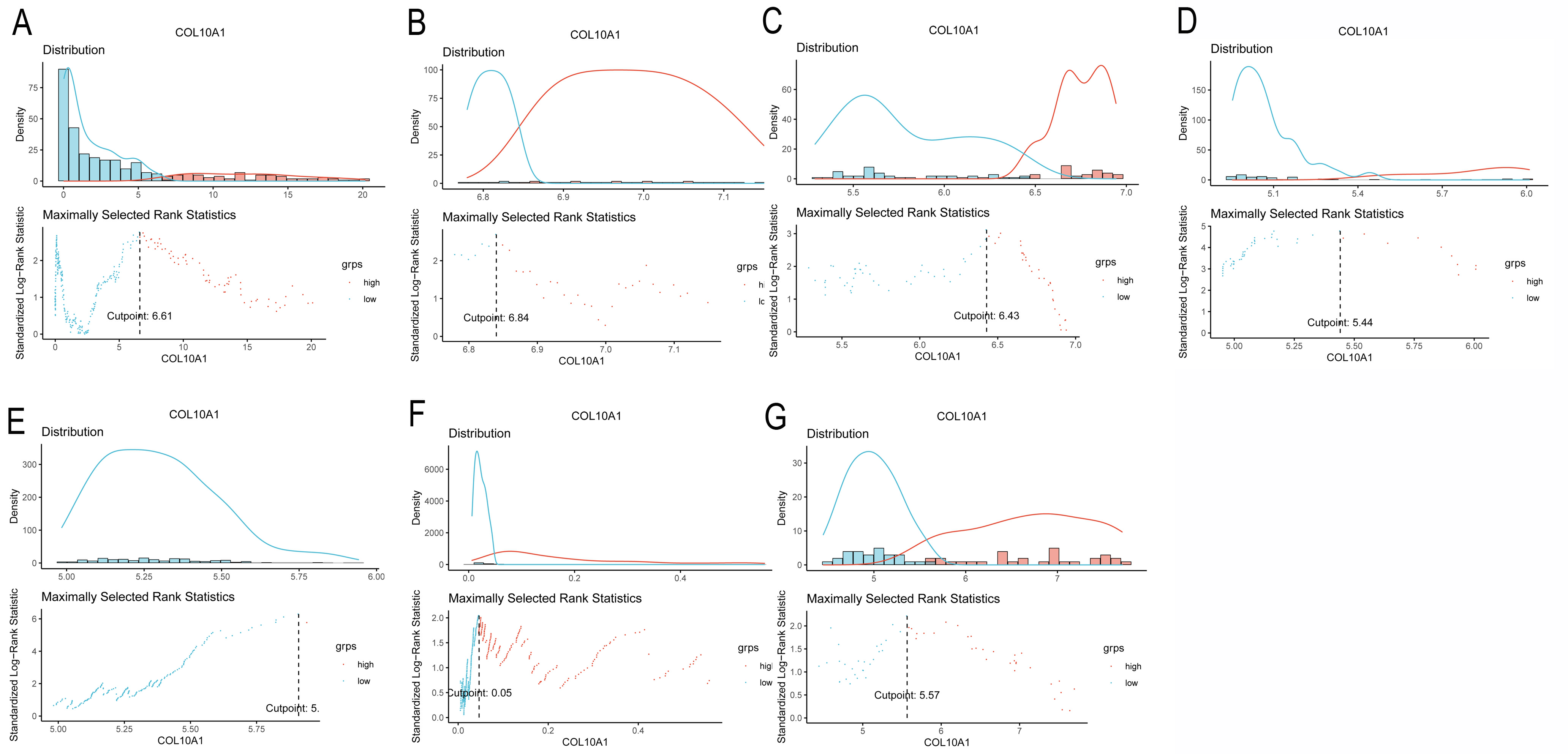

Supplement: Supplementary Figure 2 — The optimal cutoff value was used to create a categorical dependent variable based on COL10A1 expression in TCGA (A), GSE13507 (B), GSE31684 (C), GSE32548 (D), GSE62894 (E), E-MTAB-4321 (F), and E-MTAB-1803 (G) datasets. [file Image_2.tif]
